# Reliability of the color measurement of resin composites using images obtained using a stereoscopic loupe

**DOI:** 10.1590/1807-3107bor-2024.vol38.0032

**Published:** 2024-05-13

**Authors:** Alexsandra Santos ALBUQUERQUE, Rafaella BRAGANÇA, Oscar Emilio PECHO, André Luis FARIA-E-SILVA

**Affiliations:** (a) Universidade Federal de Sergipe – UFS, Dental School, Department, Aracaju, SE, Brazil.; (b) Universidade Federal de Sergipe – UFS, Graduate Program in Dentistry, Aracaju, SE, Brazil.; (c) Faculdade Meridional – IMED, School of Dentistry, Graduate Program in Dentistry, Passo Fundo, RS, Brazil.; (d) Universidade Federal de Sergipe – UFS, Graduate Program in Dentistry, Aracaju, SE, Brazil.

**Keywords:** Color, Composite Resins, Esthetics, Dental, Dentistry. Spectrophotometry

## Abstract

This study assessed the reliability of a color measurement method using images obtained from a charge-coupled device (CCD) camera and a stereoscopic loupe. Disc-shaped specimens were created using the composite Filtek Z350 XT (shades DA1, DA2, DA3, and DA4) (n = 3). CIELAB color coordinates of the specimens were measured using the spectrophotometer SP60 over white and black backgrounds. Images of the same specimens were taken using a CCD camera attached to a stereoscopic loupe. The color of the image was measured (red–green–blue [RGB]) using an image processing software and converted to CIELAB coordinates. For each color coordinate, data from images were adjusted using linear regressions predicting those values from SP60. The whiteness index for dentistry (WI_D_) and translucency parameter (TP_00_) of the specimens as well as the color differences (ΔE_00_) among pairwise shades were calculated. Data were analyzed via repeated-measures analysis of variance and Tukey’s post hoc test (α = 0.05). Images obtained using the loupe tended to be darker and redder than the actual color. Data adjustment resulted in similar WI_D_, ΔE_00_, and TP_00_ values to those observed for the spectrophotometer. Differences were observed only for the WI_D_ of shade DA3 and ΔE_00_ for comparing DA1 and DA3 over the black background. However, these differences were not clinically relevant. The use of adjusted data from images taken using a stereoscopic loupe is considered a feasible method for color measurement.

## Introduction

Color science is an important topic in the field of dentistry because several dental procedures involve esthetics, including tooth bleaching as well as enamel microabrasion and restoration. The ultimate color of the teeth depends on the interactions among optical phenomena, such as reflectance, diffraction, absorption, and transmittance of light.^1^ However, color perception by the human eyes involves subjective aspects influenced by factors such as the experience of evaluators, surrounding color, and evaluation time.^2-4^ Thus, instrumental methods using spectrophotometers or spectroradiometers are preferably used for color analysis studies. While a spectroradiometer measures absolute spectral irradiance, a spectrophotometer assesses the spectral reflectance and transmittance of a colored object.^5^ These devices usually provide color coordinates based on systems established by the International Commission on Illumination (CIE—Commission Internationale de L’Eclairage), which allows for the establishment of color differences between two objects numerically.

The human eyes possess three types of cone cells for color perception according to the sensitivity of the visual wavelength light: L-cones, sensitive to long wavelengths; M-cones, sensitive to medium wavelengths; and S-cones, sensitive to small wavelengths.^6^ Thus, several color systems are based on the tristimulus color (e.g., red–green–blue [RGB]). The CIELAB color space, which is based on lightness axis (coordinate L*) and chromatic coordinates a* (from red to green) and b* (from yellow to blue), is the most common system used in dentistry.^7,8^ Using this system, a color difference can be determined with the formula CIE76, where the CIELAB color difference formula (ΔE*_ab_) is calculated by summing the modulus of differences for all color coordinates.^8^ However, because the CIELAB color space is not perceptually uniform, the CIEDE2000 color difference formula (ΔE_00_) is currently advocated to solve this problem.^9,10^ In addition, to the accuracy of spectrophotometers, most of these devices provide large reading areas to average the surface color. However, some studies require distinguishing the color of different small areas in the same specimen. For instance, most spectrophotometers do not allow measurement of the color difference between fluorotic strips and that observed in the sound surrounding enamel.

At present, digital methods based on imaging systems and software allow determining the color of an object. The use of DSLR cameras combined with a standardized white balance gray card, which is not a validated method, has been adopted to measure tooth color in dentistry.^11,12^ However, the accuracy of color reading using this method depends on camera setting adjustment, proper calibration of white balance, and ambient lighting.^12,13^ A previous study that evaluated tooth bleaching in the presence of metallic orthodontic brackets used images obtained by a charge-coupled device (CCD) and a stereoscopic loupe to measure possible color heterogeneity in bleached tooth tissues.^14^ The color was compared under and around the bracket using the color system RGB, which does not allow for the calculation of overall color difference. This problem could be solved by the conversion of these RGB data in CIELAB color coordinates. However, this conversion is not simple, and some parameters such as illuminant (CIE standard illuminant D65) and observer angle (CIE 2°) should be considered.^15^ One solution is to calibrate or adjust the color parameters using known data obtained using a spectrophotometer.^16^ Furthermore, open-access software and linear regressions can facilitate the use of this proposed method in color evaluations in dentistry.

Therefore, the purpose of this study was to evaluate the reliability of a color measurement method. The method involved acquiring images using a CCD and a stereoscopic loupe, utilizing an open-access image processing software, and comparing the adjusted data obtained from these sources with data obtained from a spectrophotometer. The hypothesis was that the adjusted data would yield CIELAB color coordinates and parameters comparable to those obtained from a spectrophotometer.

## Methodology

### Specimen preparation

Disc-shaped specimens (diameter, 20 mm thickness, 1.6 mm) of the nanofilled resin composite Filtek Z350 XT (3M ESPE, St. Paul, MN, USA) were created by inserting a single increment into a customized silicone matrix between two polyester strips. The composite was light-activated using the light-curing unit Optilight Max (1,130 mW/cm^2^; Gnatus, Barretos, Brazil) with four 40s photoactivations (160s in total). The position of the light-curing unit tip (internal Ø ≈ 7.4 mm) was modified between each photoactivation step to cover the entire specimen surface in overlapping expositions. All specimens were carefully checked to avoid surfaces with porosities, scratches, or any defects that could affect the color measurements. No further polishing procedure was performed because the polyester strips resulted in flat and smooth surfaces. Three specimens were created for shades DA1, DA2, DA3, and DA4, which total to 12 specimens. All specimens were stored under a dry condition for at least 24 h before the color measurements.^17^

### Reference color measurements

The color of the specimens was measured (triplicate) using a spherical spectrophotometer (SP60, X-Rite, Grand Rapids, USA) in the reflectance mode, and the average values were used. The illuminating/measuring configuration was CIE d/0º, and the CIELAB color coordinates were calculated using the CIE D65 standard illuminant and 1931 2° Supplementary Standard observer. The specimens were placed against the white (L* = 92.6, a* = 1.0, and b* = −0.5) and black (L* = 32.6, a* = 1.1, and b* = 3.5) backgrounds (ColorChecker Grayscale, X-Rite, Grand Rapids, USA). No coupling agent was placed between the specimen and backgrounds.^18,19^

### Color measurement of specimen images

Images from the specimens were also taken using a CCD camera (Axiocam ERc 5s, Zeiss, Thornwood, USA) attached to a stereoscopic loupe (Zeiss Stemi 2000-C, New York, USA). The specimens were illuminated with a tungsten-halogen lamp. The camera was operated in the “continuous” mode, ensuring an automatic exposure time and automatic gain control. Snapshot resolution was defined at 2,560 × 1,920 pixels (4:3 aspect ratio). Images were captured with the specimens placed over the same backgrounds described above and recorded in the jpg format. The color of the specimen images was measured using ImageJ (NIH, Bethesda, USA), an open-source image processing software. A round region of interest of 8 mm in diameter was defined in the center of the specimens. The color of this area was measured using the “RGB measurement” plugin. The primarily defined RGB values were converted into CIELAB coordinates using MS excel spreadsheet based on the EasyRGB software (Logicol S.l.r., Trieste, Italy). Before obtaining the CIELAB values, RGB data must be converted for the CIE 1931 XYZ color space. Thus, the XYZ values of reference are used for the calculation based on observed and illuminant conditions determined in the study. The conversion was performed using X = 95,047, Y = 100,000, and Z = 108,883 as reference values, considering a 1931 2° supplementary standard observer and the CIE D65 standard illuminant.^20,21^

### Coordinate adjustment

Linear regressions were adopted to predict the values of each coordinate measured using a spectrophotometer based on those obtained from the images. Regressions were split by composite shade to determine whether this factor affects the precision of the regression models to fit the data. Because the color coordinates are significantly affected by the composite shade, pooling data is expected to result in an overestimated coefficient of concordance due to cluster effects as both methods ranked the chromaticity and lightness of shades in the same ordering. For each CIELAB color coordinate, raw data obtained from images were inserted into regression equations an “x” value, and the resulting “y” value was defined as the adjusted value. Adjusted data were used to calculate the whiteness index for dentistry (WI_D_), color difference (ΔE_00_), and translucency parameter (TP_00_), and these estimated outcomes were compared with those calculated using unadjusted data and those obtained using the spectrophotometer.

### Whiteness index

The WI_D_ of each specimen was calculated using the following equation:^22^


$$\text{ WID }=0.551\times L^{*}-2.324\times a^{*}-1.1\times b^{*}$$ Equation 1

The WI_D_ was calculated only for data obtained over the black background, considering that the equation was developed using this background color.

### Color difference among shades

The overall color difference values among the composite shades were calculated with the samples placed over black and white backgrounds using the CIEDE2000 color difference formula, according to the following equation:^9^


$$\Delta E_{00}=\sqrt{{\left(\frac{\Delta L^{\prime }}{K_{L}S_{L}}\right)}^{2}+{\left(\frac{\Delta C^{\prime }}{K_{C}S_{C}}\right)}^{2}+{\left(\frac{\Delta H^{\prime }}{K_{H}S_{H}}\right)}^{2}+R_{T}\frac{\Delta C^{\prime }}{K_{C}S_{C}}\frac{\Delta H^{\prime }}{K_{H}S_{H}}}$$ Equation 2

Being ΔL’, ΔC’, and ΔH’ the changes in luminosity, chroma, and hue, respectively. S_L_, S_C_, and S_H_ are the weighted functions for each component. K_L_, K_C_, and K_H_ are the weighted factors for lightness, chroma, and hue, respectively (K_L_ = K_C_ = K_H_ = 1). R_T_ is the interactive term between chroma and hue differences. The difference values were calculated between the colors for the same background.

### Translucency parameter

The TP_00_ based on the CIEDE2000 color difference formula (TP_00_) were calculated based on the colors of the same sample measured over the white and black backgrounds.^23^ Equation 3 was used for this purpose.


$$\Delta TP_{00}=\sqrt{{\left(\frac{\Delta L^{\prime }}{K_{L}S_{L}}\right)}^{2}+{\left(\frac{\Delta C^{\prime }}{K_{C}S_{C}}\right)}^{2}+{\left(\frac{\Delta H^{\prime }}{K_{H}S_{H}}\right)}^{2}+R_{T}\frac{\Delta C^{\prime }}{K_{C}S_{C}}\frac{\Delta H^{\prime }}{K_{H}S_{H}}}$$ Equation 3

The components of the equation were the same as previously described in Equation 2.

### Statistical analysis

The data of color coordinates (for unadjusted data), WI_D_, ΔE_00_, and TP_00_ were tested for normal distribution using the Shapiro–Wilk test and for sphericity using the Mauchly W, Greenhouse–Geisser, and Huynh–Feldt tests. For ΔE_00_ and color coordinates, the data calculated for each background were individually analyzed via repeated-measures analysis of variance (ANOVA). The same analysis was employed for the WI_D_ and TP_00_ data. The independent variables for all analyses were “composite shade” and “measurement method,” which was defined as repeated measure factor. Pairwise comparisons were performed using Tukey’s post hoc test. A confidence level of 95% was preset for all analyses, which were conducted using the open statistical platform Jamovi 1.6.15 (www.jamovi.org).

## Results

### CIELAB color coordinates

The color coordinates, obtained either directly from the spectrophotometer or calculated from the RGB values using a method that involves acquiring images through a CCD, a stereoscopic loupe, and an open-access image processing software, are presented in Table 1. Irrespective of the background color, the images tended to be darker (lower L*) and redder (higher a*) than the true color measured using the spectrophotometer. For coordinate b*, differences between the methods were observed only in the black background when the use of the spectrophotometer resulted in higher values (except for shade A4). However, despite the differences in the values, a similar ranking among the shades was found for both methods. Equations of linear regressions split according to the background neutral colors were used to adjust the CIELAB color coordinates obtained using the method presented in the current study from the data obtained using the spectrophotometer (Table 2).

**Table 1:** Means and standard deviations of the CIELAB color coordinates measured using the spectrophotometer or calculated based on a method associating images acquisition by a CCD, a stereoscopic loupe, and an open-access image processing software, for different backgrounds and composite shades.

Color coordinate	Background	Method	Composite shade			

			DA1	DA2	DA3	DA4
L*	White	Spectrophotometer	81.3 (0.4) ^Aa^	77.9 (0.2) ^Ab^	73.9 (0.5) ^Ac^	67.6 (0.2) ^Ad^
		CCD + Loupe	71.2 (0.5) ^Ba^	68.9 (0.5) ^Bb^	65.0 (0.5) ^Bc^	59.4 (0.6) ^Bd^
	Black	Spectrophotometer	78.4 (0.1) ^Aa^	75.0 (0.3) ^Ab^	71.5 (0.3) ^Ac^	65.3 (0.1) ^Ad^
		CCD + Loupe	67.4 (0.5) ^Ba^	64.7 (0.8) ^Bb^	61.8 (0.2) ^Bc^	55.8 (0.3) ^Bd^
a*	White	Spectrophotometer	0.4 (0.0) ^Bd^	2.3 (0.1) ^Bc^	3.6 (0.1) ^Bb^	4.2 (0.1) ^Ba^
		CCD + Loupe	2.3 (0.2) ^Ad^	5.1 (0.1) ^Ac^	6.7 (0.5) ^Ab^	7.6 (0.1) ^Aa^
	Black	Spectrophotometer	-1.6 (0.1) ^Bd^	-0.4 (0.1) ^Bc^	0.7 (0.1) ^Bb^	1.3 (0.1) ^Ba^
		CCD + Loupe	-1.2 (0.2) ^Ad^	1.1 (0.4) ^Ac^	2.6 (0.1) ^Ab^	4.1 (0.1) ^Aa^
b*	White	Spectrophotometer	17.1 (0.5) ^Ac^	20.6 (0.2) ^Ab^	22.5 (0.5) ^Aa^	23.8 (0.1) ^Aa^
		CCD + Loupe	16.1 (0.4) ^Ac^	20.3 (0.8) ^Ab^	21.3 (0.5) ^Ab^	23.9 (0.3) ^Aa^
	Black	Spectrophotometer	12.8 (0.3) ^Ad^	16.1 (0.4) ^Ac^	18.5 (0.3) ^Ab^	20.1 (0.2) ^Aa^
		CCD + Loupe	9.3 (0.4) ^Bd^	13.7 (0.9) ^Bc^	16.8 (0.5) ^Bb^	20.1 (0.2) ^Aa^

For each color coordinate vs. background, distinct letters (uppercase comparing methods, lowercase comparing composite shade) indicate statistical difference at Tukey`s test (p < 0.05).

**Table 2:** Coefficients (standard error) defining the equation of linear regressions which were used to adjust data for the CIELAB color coordinates obtained by a method associating images acquisition by a CCD, a stereoscopic loupe, and an open-access image processing software.

Color coordinate	Background	Equation of linear regressions
L*	White	$y=0.98(3.05)+1.12(0.05{)}^{*}x$
	Black	$y=3.54(2.58)+1.11(0.04{)}^{*}x$
a*	White	$y=-1.27(0.15)+1.71(0.03{)}^{*}x$
	Black	$y=-0.98(0.06)+0.57(0.02{)}^{*}x$
b*	White	$y=3.35(1.26)+0.87(0.06{)}^{*}x$
	Black	$y=6.57(0.33)+0.69(0.02{)}^{*}x$

In the equation, the coordinate measured with the spectrophotometer (dependent variable - y) is predicted using data calculated from images obtained with CCD + loupe (covariate – x).

CCD: charge-coupled device.

### Adjusted CIELAB color coordinates

**Figure 1:**
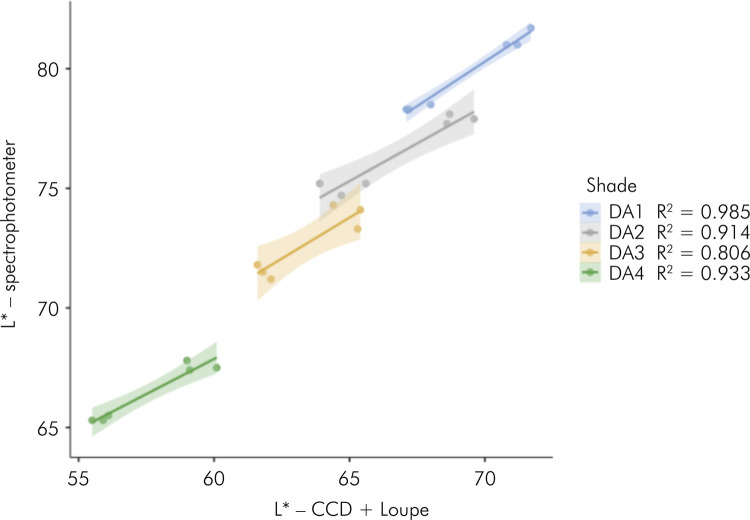
Scatterplots with regression lines (standard error) calculated with data of color coordinate L* (lightness) and split by the composite shade.

**Figure 2:**
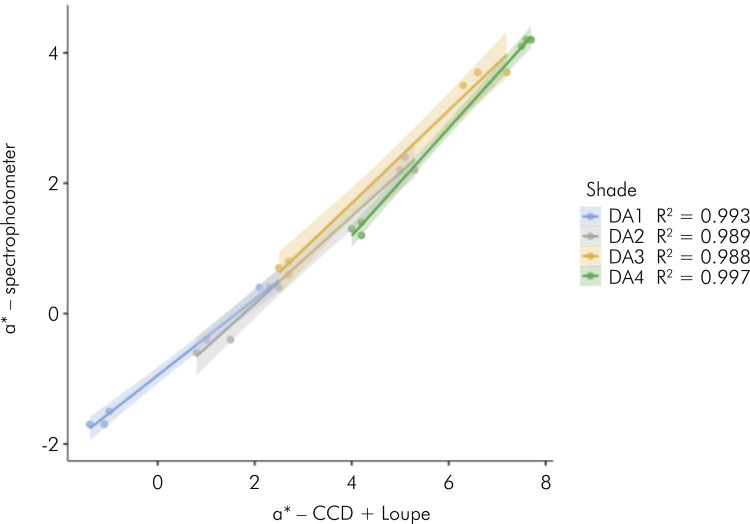
Scatterplots with regression lines (standard error) calculated with data of color coordinate a* (red-to-green axis) and split by the composite shade.

**Figure 3:**
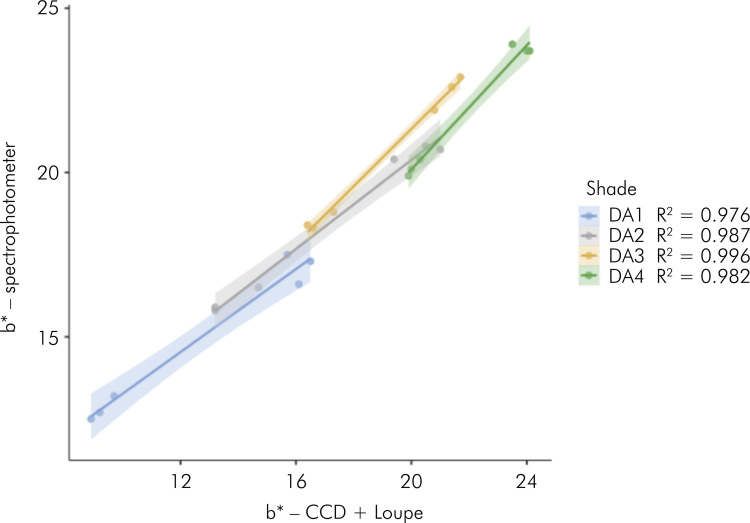
Scatterplots with regression lines (standard error) calculated with data of color coordinate b* (yellow-to-blue axis) and split by the composite shade.

Figures 1 to 3 present the results of linear regressions, illustrating the color coordinates obtained from both evaluated methods, with data segregated by composite shades. The analysis revealed high coefficients of determination for L*, ranging from 0.806 for DA2 to 0.985 for DA1. Furthermore, strong correlations were observed for the coordinates a* (ranging from 0.988 to 0.997) and b* (ranging from 0.976 to 0.996).

### Whiteness index

**Figure 4:**
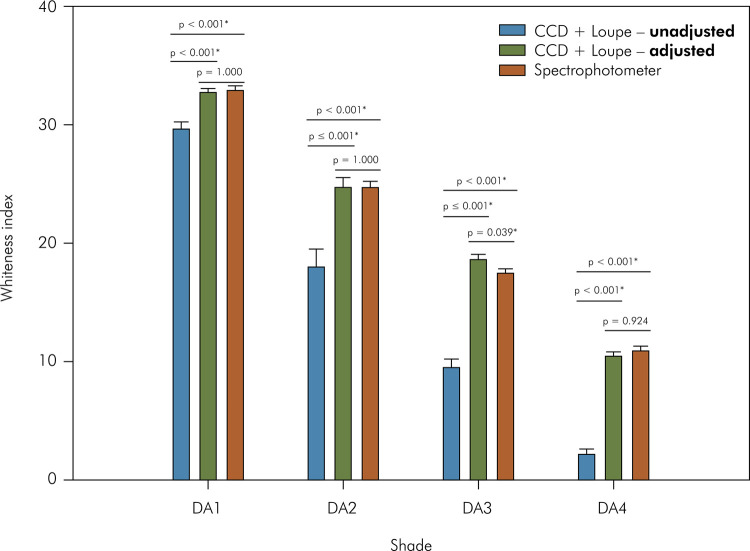
Means and standard deviations of whiteness index for dentistry (WID) measured with the spectrophotometer or calculated using images from CCD + Loupe and adjusted or not with the linear regressions. CCD: charge-coupled device. * Indicates statistical difference.

Both factors, “measurement method” (p < 0.001) and “shade” (p < 0.001), affected the WI_D_ values, and the interaction between them was significant (p < 0.001) (Figure 2). The lowest values were observed for unadjusted data obtained using the methodology proposed in the present study, irrespective of the composite shade. Similar behavior of the WI_D_ values was observed between the unadjusted data obtained using the CCD + loupe and spectrophotometer, except for A3 (lower values for the spectrophotometer). For all devices, shade DA1 had the highest WI_D_ values, followed by DA2, and the lowest values were observed for DA4 (Figure 4).

### Color differences among the composite shades

**Figure 5:**
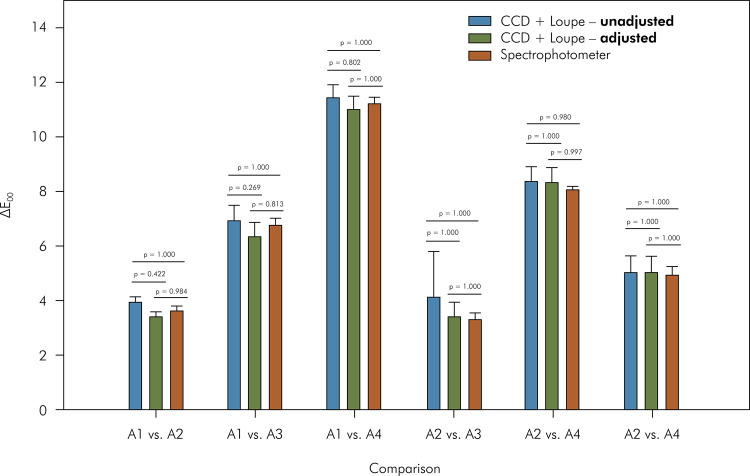
For the white background, means and standard deviations of overall color difference (ΔE00) among the color shades measured with the spectrophotometer or calculated using images from CCD + Loupe and adjusted or not with the linear regressions. CCD: charge-coupled device. * Indicates statistical difference.

**Figure 6:**
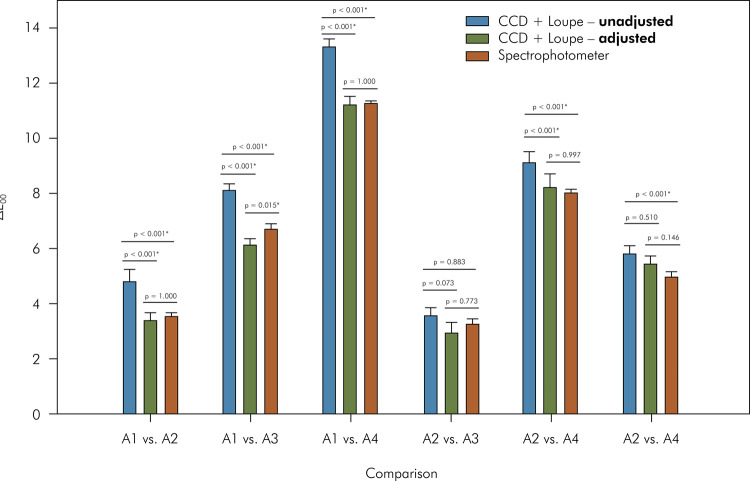
For the black background, means and standard deviations of overall color difference (ΔE00) among the color shades measured with the spectrophotometer or calculated using images from CCD + Loupe and adjusted or not with the linear regressions. (A) White background; and (B) black background. CCD: charge-coupled device. * Indicates statistical difference.

When the white background was used (Figure 5), only the independent variable “comparison” (p < 0.001) affected the ΔE_00_ values. Both the independent variable “measurement method” (p = 0.322) and the interaction (p = 0. 072) were insignificant. Irrespective of the method, A1 vs. A4 > A2 vs. A4 > A1 vs. A3 > A3 vs. A4 > A1 vs. A2 = A2 vs. A3. In contrast, both independent variables (p < 0.001) and the interaction between them (p < 0.001) were significant when the black background was used (Figure 6). Only for the comparison A1 vs. A3 was a difference between the adjusted data obtained using the CCD + loupe and spectrophotometer (higher values) observed. In general, unadjusted data obtained using the CCD + loupe yielded the highest ΔE_00_ values. However, these values were not statistically significant for the comparisons A2 vs. A3 and A3 vs. A4 (only for adjusted data). All methods had the same ranking of ΔE_00_ values: A1 vs. A4 > A2 vs. A4 > A1 vs. A3 > A3 vs. A4 > A1 vs. A2 and A2 vs. A3. However, statistical difference between the last two comparisons (A1 vs. A2 > A2 vs. A3) was observed only for unadjusted data obtained using the CCD + loupe.

### Translucency parameter

**Figure 7:**
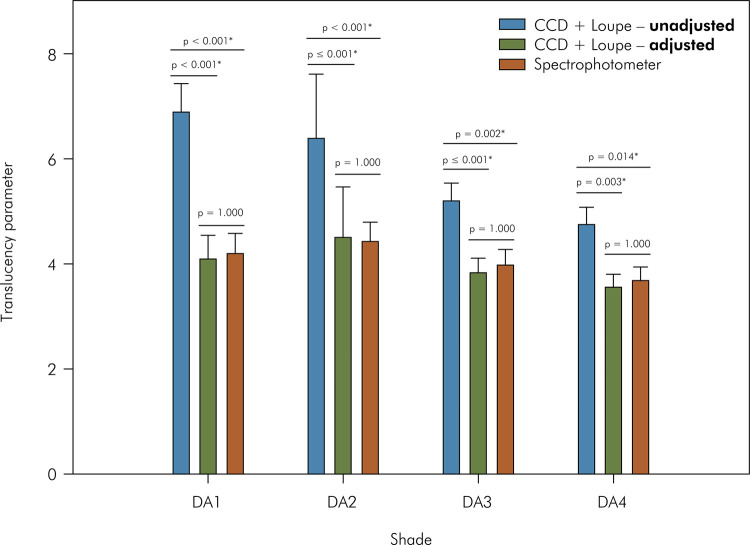
Means and standard deviations of translucency parameter (TP00) measured with the spectrophotometer or calculated using images from CCD + Loupe and adjusted or not with the linear regressions. CCD: charge-coupled device; NSD: non-significant difference. * Indicates statistical difference.

RM ANOVA revealed that only the “measurement method” (p < 0.001) affected the TP_00_ values. The p-value calculated for the independent variable “shade” (p = 0.071) was insignificant, but it was for the interaction (p < 0.001). (Figure 7). For all shades, the use of unadjusted data to calculate TP_00_ yielded the highest values, without difference between the other methods. Differences among the shades were observed only for unadjusted data obtained using CCD + loupe (A1 > A4).

## Discussion

The color determination of an object depends on three main factors, namely, the illuminant, observer, and object itself.^5^ The present study proposed the determination of the color of dental composites utilizing images taken using a CCD camera attached to a stereoscopic loupe. Unlike the spectrophotometer, it was impossible to set up a CIE standard illuminant D65 and a CIE observer angle of 2° during image acquisition.^20,21^ Illumination of the specimens on the stereoscopic loupe was provided by a tungsten-halogen lamp. Regarding the observer angle, the CIE standardizes the color readings at either 2° or 10°.^20^ The angle used is important to determine the diameter of the area analyzed. The use of a 2° observer angle at a distance of 50 cm from the object results in the visualization of an area with a 1.7 cm diameter. The diameter area would be 8.8 cm for a 10° observer angle. The observer angle is also unknown using stereoscopic loupe to obtain images. Thus, as expected, the use of the two methods analyzed in the present study resulted in significant different color coordinates for the same specimens.

Regarding lightness, the images obtained using CCD + loupe were darker (lower L* values) than the true color of the specimens. This reduction in L* values was more pronounced when the specimens were photographed over a black background. When the white background was used, linear regressions revealed that the L* values measured using the spectrophotometer were approximately one unit (interception = 0.98) higher than those calculated from images obtained using the loupe. When the black background was used, this difference increased to more than 3.5 units on average. It is reliable to assume that the use of the tungsten-halogen lamp (used as an illuminant on the stereoscopic loupe) results in less visible light reaching the specimens than when using the spectrophotometer. Different from illuminant D65, the spectrum of tungsten-halogen lamps is mainly located in the infrared with relatively reduced power in the region of the visible light spectrum.^5^ Despite the different L* values, it is crucial to emphasize that for both devices, the values changed at similar rates regardless of the background color (slopes ≈ 1.0). Another important observation can be done when linear regressions were split by the composite shade. Different from other shades (R^2^ ranging from 0.914 to 0.985), a lower coefficient of determination (≈0.80) was observed for shade DA3, indicating a lesser accurate adjustment of coordinate L* for this shade than for the others.

Regarding the chromatic coordinates, images obtained using the CCD + loupe had increased redness (higher a* values) and reduced yellowness (lower b* values) compared with the true color of the specimens. Furthermore, these differences were more pronounced when the background increased the redness (white) and reduced the yellowness (black). CIE classifies the tungsten-halogen lamp as illuminant A, and its visible spectrum continuously increases from blue to red.^5,23^ The relative spectral power at the red spectrum (620–750 nm) for illuminant A increased from 143 to 227, which is higher than that observed for illuminant D65 (reduced from 88 to 64).^5^ This fact can explain the increased redness seen in images obtained using the CCD + loupe. Consequently, illuminant D65 had lower (96–88) relative spectral power at the yellow spectrum (570–590 nm) than illuminant A (107–121)^5^. Although a yellower color would be expected for illuminant A than for D65, the opposite occurred in the present study. A reliable explanation could be attributed to the reduced irradiance of the tungsten-halogen lamp owing to its long distance from the specimens. Thus, a lower irradiance on the yellow spectrum would reach the specimens placed under the tungsten-halogen lamp as the difference in the relative spectral power between the two illuminants is smaller than that observed at the red spectrum. However, the linear regression models for the chromatic coordinates almost had perfect coefficients of determination (R^2^ ranging from 0.952 to 0.997), indicating that the behaviors of coordinates a* and b* as a function of the composite shade and background color are similar for both methods used to determine the color of the specimens.

Indeed, adjustment of the color coordinates with the linear regression models resulted in similar color measurements of the specimens for both methods, as observed when the WI_D_ was calculated. A single difference (shade DA3) between the methods was also detected. Under a black background (only used for the WI_D_ calculation), the use of adjusted data obtained from images resulted in a whiter color compared with those measured using the spectrophotometer. Interestingly, a lower coefficient of determination was observed for shade DA3 for coordinate L*; this result could be explained by a poor adjustment for the lightness. However, it is noteworthy that the difference observed between the two methods in determining the average WI_D_ of shade DA3 was 1.1, which such lower than the 50:50% acceptability threshold for whiteness index (2.62 ΔWI_D_ units) determined previously^24^. In addition, this difference was between the whiteness threshold values for “no difference” (0.70 ΔWI_D_ units) and “small difference” (1.57 ΔWI_D_ units).^24^ These results indicated that the method used in the current study of adjusting the color data from images obtained using the CCD + loupe and linear regressions accurately estimated the true color of the specimens.

An interesting usage for images obtained using the stereoscopic loupe would be to calculate color differences on specific areas of the specimens. It was previously shown that the color difference perceived by an observer is significantly affected as a function of the background color.^25^ Thus, the ΔE_00_ values were calculated using data measured against black and white backgrounds; interestingly, the results among the methods differed as a function of background. No difference was observed between the methods (including unadjusted data) for the pairwise color difference among the composite shades when using the white background. In general, the use of unadjusted data yielded the ΔE_00_ highest values. Contrarily, the ΔE_00_ values calculated with adjusted data obtained using the CCD + loupe differed from those obtained using the spectrophotometer alone when shades DA1 and DA3 were compared over the black background. Only composite shade DA3 measured over the black background had its WI_D_ values affected by the method adopted to measure the color of the specimen. However, the less accurate determination of the DA3 color using images from loupe alone intervened in the color difference calculation for the comparison involving this shade, which had a higher difference (mean ΔE_00_ of 6.72 and 6.13 units for the spectrophotometer and adjusted CCD + loupe, respectively). It is noteworthy that the difference between the methods (0.59 units) is lower than the ΔE_00_ value for the 50%:50% perceptibility threshold.^26^ Finally, unlike unadjusted data (higher TP values), the use of adjusted data from images obtained using the loupe to determine the color of the specimens resulted in similar TP_00_ values to those calculated using data from the spectrophotometer. Furthermore, as expected, no difference in the TP_00_ values among the composite shades was observed because all these shades exhibited translucency corresponding to the dental dentine.

Color measurements utilizing images obtained using a stereoscopic loupe are proposed here to evaluate color differences between small areas (e.g., spots of enamel hypoplasia), which are shorter than the measuring areas of a spectrophotometer, and the surrounding structure. As demonstrated in this study, the method of associating images from a loupe with an open-source image processing software seems to be feasible. However, it is necessary to adjust the color coordinates recorded for discrepancies with those measured using the spectrophotometer. Therefore, the hypotheses of this study were validated.

The findings of this study indicate that the use of specimens exhibiting a homogeneous color, such as ceramics or composites, allows proper adjustment of data, resulting in reliable results. Furthermore, despite color differences, the strong correlations between the color coordinates calculated using the images and those measured using the spectrophotometer allowed the use of the equations provided by linear regressions to properly adjust these data and accurately estimate color differences. It is important to emphasize that the linear regression equations described in this study should not be used for other experimental conditions. Evaluating other materials or obtaining images under different conditions (e.g., illuminant) can affect color coordinates, and these coordinates should be adjusted for each experimental condition. Further studies are warranted to determine whether the same adjustment can be effective using other dental materials and shades.

## Conclusions

This study demonstrated that the use of images obtained using the stereoscopic loupe to digitally measure the color of the specimens was a reliable method. However, the color coordinates need to be adjusted utilizing data obtained using a spectrophotometer.
